# Comparison of Environmental DNA Metabarcoding and a Traditional Survey Method for Assessing Fish Diversity and Distribution Along Salinity Gradient in an Urban Brackish Reservoir, China

**DOI:** 10.3390/biology13110930

**Published:** 2024-11-15

**Authors:** Xu Wang, Jiaqiao Wang, Lin Lin, Liangmin Huang, Kai Liu, Guangjie Dai, Qianwen Cai, Jun Li, Shilong Feng, Guangzhao Wang, Yapeng Hui, Longhui Qiu, Fenfen Ji

**Affiliations:** 1 Fisheries College, Jimei University, Xiamen 361021, China; 202412951039@jmu.edu.cn (X.W.); skyofstar1@jmu.edu.cn (J.W.); 202411710015@jmu.edu.cn (L.L.); lmhuang@jmu.edu.cn (L.H.); liukai1218@jmu.edu.cn (K.L.); lijun1982@jmu.edu.cn (J.L.); 202312951064@jmu.edu.cn (S.F.); 202221063031@jmu.edu.cn (G.W.); 202412951041@jmu.edu.cn (Y.H.); 2 Agriculture, Rural and Water Resources Bureau of Jimei District, Xiamen 361022, China; dgjdgj14@sina.com (G.D.); baicai77@sina.com (Q.C.); 3 Engineering Research Center of Green Development for Conventional Aquatic Biological Industry in the Yangtze River Economic Belt, Ministry of Education, College of Fisheries, Huazhong Agricultural University, Wuhan 430070, China; longhui@webmail.hzau.edu.cn

**Keywords:** fish diversity, environmental DNA metabarcoding, traditional survey method, Xinglinwan Reservoir

## Abstract

With the decrease in fish diversity, the monitoring of fish has become a current research hotspot. Here, the fish diversity and spatial distribution along a salinity gradient (0.28‰–3.32‰) in Xinglinwan Reservoir, China, were investigated using eDNA and the TSM. With the combination of both methods, 12 species from 6 orders, 9 families, and 11 genera were observed. The invasive species *Oreochromis niloticus* had the highest relative abundance, accounting for 56.91% in the TSM and 89.80% in eDNA, respectively. eDNA detected 83.33% of the species detected using the TSM, and an additional six species (50.0% of the total species) were detected via only eDNA. Moreover, eDNA supplemented the unavailable abundance data from the TSM. The TSM revealed that the diversity and relative abundance of fish inhabiting seawater and brackish water increased with an increase in salinity, but this was not found using eDNA. Thus, our study highlights that Xinglinwan Reservoir has a low species richness and is suffering from the great harm of ecological invasion. Our findings also show that eDNA could be reliable for providing a comprehensive picture of fish, but discriminating the spatial heterogeneity of fish communities is a challenge for its widespread adoption in lentic brackish systems.

## Simple Summary

Urbanization has been regarded as a major threat to fish diversity in urban waters. Xinglinwan Reservoir, a lentic brackish ecosystem with a distinct salinity gradient in southeastern China, is experiencing rapid urbanization, and there are no reports on fish diversity here. Environmental DNA metabarcoding (eDNA) has been applied to biodiversity assessments in aquatic ecosystems, but there has been limited work on fish diversity in lentic brackish ecosystems. We assessed the fish diversity and spatial distribution along a salinity gradient in Xinglinwan Reservoir, combining eDNA and the traditional survey method (TSM). This study provides realistic pictures of fish species assemblages in Xinglinwan Reservoir and tests the efficiency of eDNA for assessing fish diversity in lentic brackish ecosystems. Our results contribute to the conservation of fish resources in Xinglinwan Reservoir and advance the application of eDNA in lentic brackish ecosystems.

## 1. Introduction

Fish are an important component of freshwater ecosystems and are particularly valuable to the fishery industry [1]. However, fish diversity is seriously threatened worldwide, mainly due to habitat degradation by anthropogenic land use, particularly urbanization, followed by other severe threats, such as pollution and invasive species [2]. The extensive loss of fish diversity calls for increased monitoring to precisely evaluate changes in fish communities and conserve fish resources.

The traditional survey method (TSM) used to assess fish diversity in the past involves morphological identification [3,4]. However, this method is time-consuming, challenging for identification purposes, and potentially harmful to fish [5]. Environmental DNA metabarcoding (eDNA) directly retrieves genetic material from environmental samples, including sediment, excreta, air, and water [6,7]. It rapidly and accurately analyzes the community structure of target groups in the environment by identifying sequence differences in specific DNA fragments between species [8]. This revolutionary approach transforms the traditional research model of species identification based on morphological characteristics and significantly enhances the efficiency of biodiversity assessment [7,9,10]. Although numerous studies have been conducted on biodiversity using eDNA, most of them have focused on assessing fish diversity in freshwater and marine environments [11,12,13,14,15]. As a result, few studies have assessed fish diversity using eDNA in brackish water, especially in lentic water bodies.

Xiamen, located in China’s special economic zone and southeast coastal area, holds great significance as a central hub, major port, and renowned tourist destination. Xinglinwan Reservoir, situated in Xiamen, is a valuable ecological, scenic, and water resource. It was designated as a National Urban Wetland Park in 2010 and was included in the initial selection of the list of important provincial wetlands in 2017 [16]. The northern region is replenished by the upstream Bantou Reservoir and the convergence of precipitation, while the southern region is influenced by the seawater from the West Sea of Xiamen, resulting in brackish water [17]. Current research on fish diversity is lacking, and Xinglinwan Reservoir has been plagued by the presence of invasive fish for a considerable period of time [16]. This study evaluates the fish community diversity using eDNA and the TSM. The objectives of this study are to (i) provide realistic pictures of fish species assemblages in Xinglinwan Reservoir and (ii) test the efficiency of eDNA in assessing fish diversity in lentic brackish waters.

## 2. Materials and Methods

### 2.1. Study Area and Sample Location

**Figure 1:**
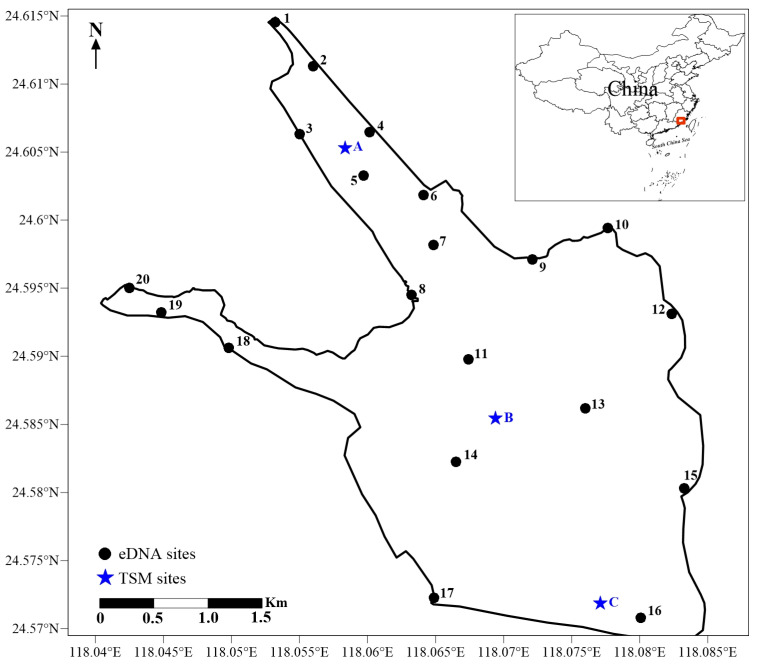
Schematic diagram of Xinglinwan Reservoir and sampling sites.

Xinglinwan Reservoir is in Xiamen city of Fujian Province, China (29°46′71″–29°51′45″ N, 112°31′36″–112°37′30″ E) and was built with the completion of Jixing dam in 1956 [16]. The dam is situated at the estuary of the Houxi River. Xinglinwan Reservoir covers an area of 6 km^2^, with an average depth of 5.5 m [16], and the salinity gradually increases from the northern freshwater inlet to the southern estuary, forming a distinct salinity gradient from A to C (the salinity at A, B, and C is 0.28‰, 1.28‰, and 3.32‰; Figure 1). Furthermore, like many coastal regions, its watershed is experiencing rapid urbanization [18]. Domestic pollutants discharged from upstream into the bay cause heavy organic pollution and eutrophication [19]. Xinglinwan Reservoir is a poorly flushed, semi-enclosed waterbody with a limited self-purification capacity [20]. The aquatic ecosystem is not favorable for the proliferation and reproductive activities of most fish species. Evaluating the fish assembles is of great significance and the premise for the conservation and recovery of fish biodiversity. Based on the variations in the salinity gradients, the availability of samples, and the representativeness of the sampling sites, the TSM was used at 3 sites and eDNA was used at 20 sites to census fish diversity in Jan 2023 in Xinglinwan Reservoir (Figure 1).

### 2.2. Sampling Collection for Traditional Method

Samples for the TSM were obtained following a standard for the investigation of reservoir fishery resources [21]. Gillnets and traps were used to collected fish samples [22]. Two gillnets of each size (50.0 m length × 1.5 m height, with uniform stretched mesh sizes of 28.0, 60.0, and 100.0 mm) and four traps (50.0 m in length and 30 cm in diameter) were set at each site. The gillnets were set perpendicular to the shoreline at logistically favorable sites for 3 h, and then the fish catch was collected at each site. The traps were set for 24 h at each site. The fish samples were stored on ice and taken back to the laboratory within 2 h. In the laboratory, the fish were identified immediately at the lowest possible taxonomic level according to the relevant references [23].

### 2.3. Sampling Collection and Analyses for eDNA

At each site, a water sample (1 L) for eDNA was collected with a water sampler from 5–10 cm below the surface and stored in a sterile plastic bottle (1 L). All samples were stored on ice until filtration. Each 1 L water sample was filtered within 4 h of collection through a 0.45 μm sterile Millipore Mixed Cellulose Membrane (JINTENG, Tianjin, China) using a filtration unit (YUZE, Tianjin, China). The filtration unit and forceps were sterilized using sodium hypochlorite and then rinsed with ddH_2_O (double-distilled H_2_O) to remove residual bleach between each site to avoid contamination. The field crews wore sterile gloves to collect and handle samples [24]. All filters were stored individually in sterile 5 mL centrifuge tubes at −20 °C until DNA extraction [1].

DNA extraction was performed using an MP FastDNA^®^ Spin Kit (MP Biomedicals, Santa Ana, CA, USA). All extracted DNA samples were stored at −20 °C until further analysis. Metabarcoding was conducted on each DNA extract with the primers Actinopterygii 16s (Ac16s)-F (5′-CCTTTTGCATCATGATTTAGC-3’) and Ac16s-R (5’-CAGGTGGCTGCTTTTAGGC-3’) [25], which target the 16S rDNA gene region of the mitochondrial genome, to identify fish species. The polymerase chain reaction (PCR) assay volume was 20 μL, including 5× TransStart FastPfu buffer, 4 μL; 2.5 mM dNTPs, 2 μL; forward primer (5 μM), 0.8 μL; reverse primer (5 μM), 0.8 μL; TransStart FastPfu DNA Polymerase, 0.4 μL; template DNA, 10 ng; and ddH_2_O to a final volume of 20 μL. For all samples, PCR was performed as follows: 94 °C for 3 min, followed by 45 cycles at 94 °C for 30 s, 60 °C for 30 s, and 72 °C for 30 s, with a final extension at 72 °C for 5 min. The amplification size was approximately 350 bp. The PCR product was extracted from a 2% agarose gel, purified using an AxyPrep DNA Gel Extraction Kit (Axygen Biosciences, San Diego, CA, USA), and quantified using a Quantus™ Fluorometer (Promega, Madison, WI, USA). The purified amplicons were pooled in equimolar amounts and paired-end sequenced on an Illumina MiSeq PE300 platform (Illumina, San Diego, CA, USA). The raw sequence reads are deposited in the NCBI Sequence Read Archive database with the BioProject ID PRJNA1162281 (https://www.ncbi.nlm.nih.gov/sra/PRJNA1162281 (accessed on 18 September 2024)).

The raw sequencing reads were demultiplexed, quality-filtered using Trimmomatic, and merged using FLASH v 1.2.7 [26] with the following criteria: (i) 300 bp reads were truncated at any site receiving an average quality score of <20 over a 50 bp sliding window, discarding the truncated reads that were shorter than 50 bp. (ii) Paired-end sequences overlapping longer than 10 bp were merged into a single sequence. (iii) The sequence direction was adjusted, the exact barcode was matched, and a maximum of two nucleotide mismatches were included in primer matching. (ⅳ) Operational taxonomic units (OTUs) with 97% similarity cutoffs were clustered using UPARSE v 7.1 [27], and chimeric sequences were removed. The taxonomy of each OTU representative sequence was analyzed using the Ribosomal Database Project (RDP) Classifier v 2.2 against the Nucleotide Sequence Database (NT) [28]. (ⅴ) Nontarget species (e.g., bacteria and viruses) were discarded [6]; for species-filtering steps, see Figure S1.

### 2.4. Community Data Collection and Analyses

#### 2.4.1. Species Richness

The species richness of each taxonomic level identified using eDNA and the TSM was calculated based on a species presence/absence table [5]. Then, for each site, the percentage of species richness detected using each method was calculated as the number of species detected using the individual method divided by the total number of species detected [6,24].

#### 2.4.2. The Relative Abundance

The relative abundances of the species were calculated from the OTU abundance table for eDNA and the species count data for the TSM [29,30]. Specifically, the relative abundance of species per site detected using eDNA was calculated as the number of sequences of the species divided by the total number of sequences [29], whereas for the TSM, it was calculated as the number of individuals of the species divided by the total number of individuals [30].

#### 2.4.3. Diversity Index

The formulas for the diversity indices are as follows:

Margalef Index:

H=(S−1)/lnN

Shannon Index:

$$H^{\prime }=-\overset{S}{\underset{i=1}{\sum }}P_{i}\ln P_{i}$$

Simpson Index:

$$D=1-\overset{S}{\underset{i=1}{\sum }}P_{i}^{2}$$

Pielou Index:

$$J=H^{\prime }/\ln S$$

For the TSM, *S* is the total number of species; *N* is the number of individuals of all species; and *P_i_* is the number of individuals of the *i*th species divided by the total number of individuals [31,32,33]. For eDNA, *S* also represents the total number of species; *N* is the total number of sequences of all species; and *P_i_* is the number of sequences of the *i*th species divided by the total number of sequences of all species [34].

#### 2.4.4. Body Length and Weight of Fish

The numbers of individual fish were counted for the TSM. The body length and weight of 30 randomly selected tail fish were measured for species with a population size exceeding 30 individuals. The body length and weight of all individuals were measured for species with a population size below 30. The body length measurement is accurate to 0.1 cm, while the weight measurement is accurate to 0.01 g.

### 2.5. Statistical Analyses

Statistical analyses were conducted with SPSS 26.0 software and Excel 2017. Pie charts of species composition, histograms depicting the relative abundance of species, and line charts of diversity per site were generated in Origin 2022.

## 3. Results

### 3.1. Community Profile in the Xinglinwan Reservoir

**Figure 2:**
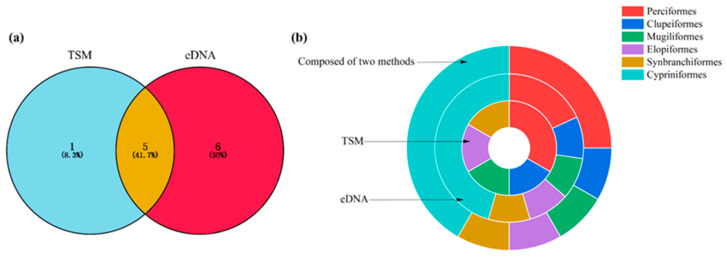
Species richness of fish identified using the TSM and eDNA (**a**). The percentage of order richness detected using eDNA, TSM, and both methods (**b**).

Using the TSM, six fish species from five orders, six families, and six genera were detected. Using eDNA, a total of 1,596,608 reads were obtained, and 11 fish species from 6 orders, 8 families, and 10 genera were identified. When the eDNA and TSM datasets were combined, 12 fish species from 6 orders, 9 families, and 11 genera were observed. Five species (41.7%) were identified through both eDNA and the TSM; additionally, six (50.0%) and one (8.3%) species were detected via eDNA and the TSM alone, respectively (Figure 2a; Table 1).

For the TSM, the order containing the greatest number of species was Perciformes (two species), accounting for 33.32%; Clupeiformes (one species), Mugiliformes (one species), Elopiformes (one species), and Synbranchiformes (one species) each accounted for 16.67% (Figure 2b). For eDNA, the order containing the greatest number of species was Cypriniformes (five species), accounting for 45.45%; Perciformes contained two species, accounting for 18.19%. Clupeiformes (one species), Mugiliformes (one species), Elopiformes (one species), and Synbranchiformes (one species) each accounted for 9.09% (Figure 2b). When the eDNA and TSM datasets were combined, the orders containing the greatest numbers of species were Cypriniformes (five species) and Perciformes (three species), accounting for 41.68% and 25.00%, respectively; Clupeiformes (one species), Mugiliformes (one species), Elopiformes (one species), and Synbranchiformes (one species) each accounted for 8.33% (Figure 2b).

### 3.2. Relative Abundance and Diversity

**Figure 3:**
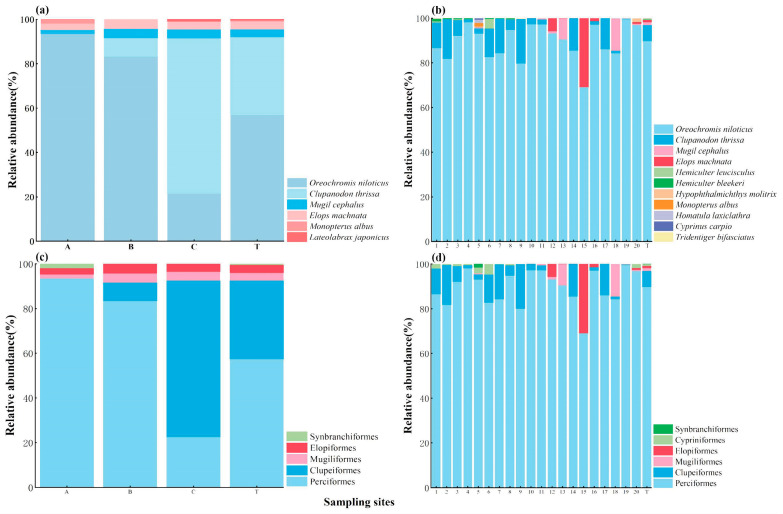
Relative abundance (%) of fish at the species level (**a**,**b**) and order level (**c**,**d**) with the TSM (**a**,**c**) and eDNA (**b**,**d**) per sampling site. The relative abundance of fish for overall reservoir scales is marked with T.

Regarding the relative abundance of fish, for the TSM, the most abundant species was *Oreochromis niloticus* (56.91%), followed by *Clupanodon thrissa* (35.13%) (Figure 3a). The relative abundance of *O. niloticus* obtained using the TSM at the A, B, and C sites was 93.46%, 83.3%, and 21.5% respectively, while that of *C. thrissa* was 0.00%, 8.30%, and 70.00%, respectively (Figure 3a). Similarly, for eDNA, the most abundant species was *O. niloticus* (89.80%), followed by *C. thrissa* (7.13%; Figure 3b). The relative abundance of *O. niloticus* obtained using eDNA was the greatest for all the sampling sites, ranging from 69.04% (site 15) to 99.57% (site 19; Figure 3b). The relative abundance of *C. thrissa* detected using eDNA ranged from 0.00% (site 15) to 19.94% (site 9; Figure 3b).

For both methods, the family with the greatest relative abundance was Cichlidae, followed by Clupeidae, accounting for 56.91% and 35.13% in the TSM (Figure S2a) and 89.80% and 7.13% in eDNA (Figure S2b). For both methods, the order with the greatest relative abundance was Perciformes, followed by Clupeiformes, accounting for 57.38% and 35.13% in the TSM (Figure 3c) and 89.82% and 7.13% in eDNA (Figure 3d).

**Figure 4:**
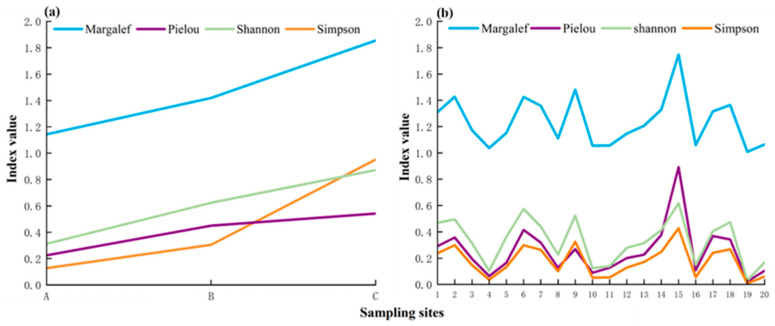
Fish diversity index per site detected using the TSM (**a**) and eDNA (**b**).

The fish diversity indices, namely, the Shannon Index, Margalef Index, Simpson Index, and Pielou Index, were assessed at each site using the TSM and eDNA. For the TSM, the Shannon Index at sites A, B, and C was 0.312, 0.624, and 0.872, respectively. The Margalef Index at sites A, B, and C was 1.143, 1.419, and 1.855, respectively. The Simpson Index at sites A, B, and C was 0.127, 0.305, and 0.951, respectively. The Pielou Index at sites A, B, and C was 0.225, 0.450, and 0.541, respectively (Figure 4a). The values of each index exhibited a gradual increase from site A to site C (Figure 4a). For eDNA, the mean value of the Shannon Index was 0.331, ranging from 0.031 to 0.619. The average value of the Margalef Index was 1.241, with a range of 1.009–1.747. The average value of the Simpson Index was 0.174, ranging from 0.009 to 0.428. The average value of the Pielou Index was 0.253, with a range between 0.022 and 0.893 (Figure 4b).

### 3.3. Body Length and Weight Characteristics of Fish

The body length and weight of each fish were analyzed based on the TSM. The average body length of *O. niloticus* was 11.91 cm, ranging from 5.00 cm to 18.40 cm, and the mean weight was 76.02 g, ranging from 4.00 g to 214.00 g. The average body length of *C. thrissa* was 17.01 cm, ranging from 14.10 cm to 18.80 cm, and the mean weight was 83.47 g, ranging from 53.00 g to 110.00 g. The average body length of *Mugil cephalus* was 27.00 cm, ranging from 19.50 cm to 30.60 cm, and the mean weight was 353.29 g, ranging from 133.00 g to 501.00 g. The average body length of *Megalops cyprinoides* was 24.15 cm, ranging from 19.20 cm to 30.70 cm, and the mean weight was 142.00 g, ranging from 73.00 g to 247.00 g. The average body length of *Monopterus albus* was 49.80 cm, ranging from 49.30 cm to 50.30 cm, and the mean weight was 106.50 g, ranging from 103.00 g to 110.00 g. The average body length of *Lateolabrax japonicus* was 30.60 cm, ranging from 21.20 cm to 40.00 cm, and the mean weight was 578.00 g, ranging from 156.00 g to 1000.00 g (Table 2).

## 4. Discussion

In this study, the fish diversity and spatial distribution along a salinity gradient in Xinglinwan Reservoir, China, were estimated using eDNA and the TSM. Our findings suggest that the species richness is low and suffering from the great harm of ecological invasion in Xinglinwan Reservoir. We also highlight that eDNA is a rapid and reliable method for providing a comprehensive picture of fish, but discriminating the spatial heterogeneity of fish communities is crucial for the widespread adoption of eDNA in lentic brackish systems.

### 4.1. Fish Community Characteristics in the Xinglinwan Reservoir

In the present study, 12 species from 6 orders, 9 families, and 11 genera were observed in Xinglinwan Reservoir (Figure 1). The species richness in Xinglinwan Reservoir was relatively poorer than that in other reservoirs, including Sanbanxi Reservoir, Xinanjiang Reservoir, and Pingzhai Reservoir [35,36,37] (Table 3). Moreover, the invasive species *O*. *niloticus* (56.91% in the TSM and 89.80% in eDNA) had the highest relative abundance in Xinglinwan Reservoir. Thus, Xinglinwan Reservoir is suffering from the great harm of ecological invasion. *O*. *niloticus* is native to Africa and is one of the most popular fishes used in aquaculture due to its ability to tolerate a wide range of environmental conditions, flexible habitat requirements, reproductive strategies, fast growth, and aggressive and omnivorous feeding habits [38,39]. When *O*. *niloticus* becomes the dominant organism, it outcompetes native species for nutrients and space within the aquatic ecosystem [38,39,40]. Additionally, *Oreochromis* Sp. may prey on the eggs of native fish, leading to the decline of the biomass of native fish species and threating fish biodiversity [40,41]. These factors could contribute significantly to the observed low fish species richness in Xinglinwan Reservoir. Previous research demonstrated that invasive *O*. *niloticu* not only affected the CPUE (catch-per-unit-per-effort) of the fish community and native fish species but also inhibited the growth of mud carp with similar prey groups in the main rivers of Guangdong Province, China [42]. Moreover, the relative abundance of *O*. *niloticus* exhibited a gradual increase over time following the invasion [43]. In particular, the established *O*. *niloticus* significantly undermined the relative densities of the native fishes [43]. Our findings further indicate that *O*. *niloticus* constitutes the predominant fish population in Xinglinwan Reservoir, characterized by a relatively small individual size (Table 4). Compared with other waters, the individual size of *O*. *niloticus* in Xinglinwan Reservoir is obviously smaller [44,45] (Table 4). Thus, our findings indicate that Xinglinwan Reservoir has a low species richness and is suffering from the great harm of ecological invasion due to invasive *O*. *niloticus* having the highest relative abundance with a small individual size.

### 4.2. Fish Community Assessment with eDNA in Lentic Brackish Waters

In the present study, 12 fish species were monitored using eDNA and the TSM, in which 5 species (41.7%) were identified through both eDNA and the TSM and an additional 6 (50.0%) and 1 (8.3%) species were detected via eDNA and the TSM alone, respectively (Figure 2). eDNA detected 83.33% of the species detected using the TSM (Table 1). Furthermore, the sampling sites for the TSM were located in open water, while the sites for eDNA included both open water and nearshore water. Thus, the fish richness results from the open water sampling sites (sites 5, 7, 11, 13, 14, and 16) were compared between the TSM and eDNA. The results show that 11 fish species were detected using eDNA and the TSM, in which 5 species (45.5%) were identified through both eDNA and the TSM and an additional 5 (45.5%) and 1 (9.1%) species were detected via eDNA and TSM alone, respectively (Figure S3). Thus, our study found that the species consistency between eDNA and the TSM was quite high, with eDNA detecting significantly more species. Previous studies also found that eDNA identified a significantly greater number of fish species than the TSM in an estuary ecosystem [5,46]. Furthermore, regarding the relative abundance of fish, the eDNA and TSM results revealed that *O. niloticus* and *C. thrissa* exhibited the highest abundance, accounting for 89.80% and 7.13% in eDNA and 56.91% and 35.13% in the TSM, respectively (Figure 3a). Similarly, the fish relative abundance results from the open water sampling sites (sites 5, 7, 11, 13, 14, and 16) were compared between the TSM and eDNA. *O. niloticus* and *C. thrissa* still showed the highest abundances, accounting for 91.34% and 5.89%, respectively. Moreover, the relative abundance of other fish determined using the TSM was found to be consistent with that detected using eDNA (Figure 3). In addition, eDNA supplemented the unavailable abundance data from the TSM (Figure 3). Similarly, a previous study showed that the relative abundance of fish detected using eDNA and the TSM demonstrated consistent seasonal variation patterns, and eDNA provided additional fish abundance data that could not be obtained through the TSM in an estuary ecosystem [5]. Therefore, our findings further highlight that eDNA can generally reflect the relative abundance of fish well in lentic brackish waters and complement abundance information unavailable using the TSM. Additionally, the TSM results show that the relative abundance of fish inhabiting seawater and brackish water increased with the rise in salinity. Specifically, using the TSM, the relative abundance of *C. thrissa* at A, B, and C was found to be 0.00%, 8.30%, and 70.00%, respectively, while this pattern was not found using eDNA (Figure 3). In addition, the values of each diversity index exhibited a gradual increase from site A to site C, while this was not exhibited by eDNA (Figure 4). Furthermore, a comparison was made between the TSM and eDNA results of the relative abundance of fish obtained from the open water sampling sites (sites 5, 7, 11, 13, 14, and 16) and all sites (including both nearshore and open water), respectively, revealing very small differences in species relative abundance for the open water sampling sites and all sites (Figure 3d and Figure S4). These results indicate that discerning the spatial heterogeneity in fish communities is still a challenge for eDNA. The available research indicates that the key to accurately assessing fish abundance using eDNA lies in quantifying the relationship between the number of fish observed using the TSM and the number of sequences obtained using eDNA [47]. In a previous study, the significant, positive relationship between traditional estimates of adult walleye populations (individuals) and eDNA concentration was estimated to monitor the fish abundance in lakes [47]. Our findings further indicate that understanding the correlation between species individuals identified using the TSM and DNA sequences identified using eDNA is a major challenge for using eDNA to evaluate fish abundance and determine the spatial distribution of fish in lentic brackish waters. Moreover, our study indicates that eDNA detects significantly more species and supplements the unavailable abundance data from the TSM; however, they also indicate that it is still a challenge for eDNA to determine the spatial heterogeneity in fish communities.

## 5. Conclusions

This study provides a realistic survey of fish diversity and spatial distribution along a salinity gradient in Xinglinwan Reservoir via eDNA and the TSM. A total of 12 species were detected, and the invasive species *O. niloticus* was found to have the highest relative abundance in Xinglinwan Reservoir. Compared with the traditional results, eDNA detected significantly more species and supplemented the unavailable abundance data from the TSM. Moreover, understanding the correlation between species individuals identified using the TSM and DNA sequences identified using eDNA is key for using eDNA to determine the spatial distribution of fish in lentic brackish waters. Overall, Xinglinwan Reservoir has a low species richness and is suffering from the great harm of ecological invasion. eDNA could be reliable for providing a comprehensive picture of fish, but discriminating the spatial heterogeneity of fish communities is a challenge for its widespread adoption in lentic brackish systems.

## Figures and Tables

**Table 1:** Species detected in Xinglinwan Reservoir using eDNA and the TSM.

Species	Genus	Family	Order	eDNA	TSM
*Oreochromis niloticus*	*Oreochromis*	Cichlidae	Perciformes	+	+
*Clupanodon thrissa*	*Clupanodon*	Clupeidae	Clupeiformes	+	+
*Mugil cephalus*	*Mugil*	Mugilidae	Mugiliformes	+	+
*Megalops cyprinoides*	*Megalops*	Megalopidae	Elopiformes	*+*	*+*
*Monopterus albus*	*Monopterus*	Synbranchidae	Synbranchiformes	+	+
*Lateolabrax japonicus*	*Lateolabrax*	Lateolabracidae	Perciformes	−	+
*Hypophthalmichthys molitrix*	*Hypophthalmichthys*	Cyprinidae	Cypriniformes	+	−
*Homatula laxiclathra*	*Paracobitis*	Nemacheilidae	Cypriniformes	+	−
*Cyprinus carpio*	*Cyprinus*	Cyprinidae	Cypriniformes	+	−
*Tridentiger bifasciatus*	*Tridentiger*	Gobiidae	Perciformes	+	−
*Hemiculter leucisculus*	*Hemiculter*	Cyprinidae	Cypriniformes	+	−
*Hemiculter bleekeri*	*Hemiculter*	Cyprinidae	Cypriniformes	+	−

Note: “−” indicates species absent from the TSM and eDNA results; “+” indicates species present in the TSM and eDNA results.

**Table 2:** Body length and body weight of fish in the Xinglinwan Reservoir.

Populations	Body Length/cm		Body Weight/g	
	Range	Mean ± S.D	Range	Mean ± S.D
*Oreochromis niloticus*	5.00–18.40	11.91 ± 3.10	4.00–214.00	76.02 ± 48.38
*Clupanodon thrissa*	14.10–18.80	17.00 ± 0.99	53.00–110.00	83.50 ± 12.63
*Mugil cephalus*	19.50–30.60	27.00 ± 2.72	133.00–501.00	353.29 ± 91.71
*Megalops cyprinoides*	19.20–30.70	24.15 ± 3.39	73.00–247.00	142.00 ± 51.70
*Monopterus albus*	49.30–50.30	49.80 ± 0.71	103.00–110.00	106.50 ± 4.95
*Lateolabrax japonicus*	21.20–40.00	30.60 ± 13.29	156.00–1000.00	578.00 ± 596.80

**Table 3:** The species richness in the Xinglinwan Reservoir and other Reservoir.

Study Area	Species	Order	Family	Genus	Reference
Xinglinwan Reservoir	14	5	9	11	This study
Sanbanxi Reservoir	48	4	12	38	[35]
Xinanjiang Reservoir	75	8	17	/	[36]
Pingzhai Reservoir	43	5	11	37	[37]

Note: “/” indicates the data could not be available from the references.

**Table 4:** Differences in body length and weight among different populations of *O. niloticus*.

Study Area	Body Length (cm)	Body Weight (g)	Reference
Xinglinwan Reservoir	11.91	76.02	This study
Shanmei Reservoir	25.30	520.00	[44]
Yalong River Ertan reservoir area	15.68	221.00	[45]

## Data Availability

The raw sequence reads detected by eDNA are deposited in the NCBI Sequence Read Archive database with the BioProject ID PRJNA1162281 (https://www.ncbi.nlm.nih.gov/sra/PRJNA1162281 (accessed on 18 September 2024)). Part of the data presented in this study are available in the Supplementary Material. The reaining data presented in this study are available upon reasonable request from the corresponding author.

## Supplementary Materials

The following supporting information can be downloaded at: https://www.mdpi.com/article/10.3390/biology13110930/s1, Table S1: species detected in the the Xinglinwan Reservoir using eDNA and TSM; Figure S1: Species-filtering steps identifying target species; Figure S2: Relative abundance (%) of fish at the family level with TSM (a) or eDNA (b) in per sampling site. Figure S3: Species richness of fish identified using the TSM and eDNA for the open water sampling sites (sites 5, 7, 11, 13, 14, and 16). Figure S4: Relative abundance (%) of fish at the species level with eDNA for the open water sampling sites (sites 5, 7, 11, 13, 14, and 16). The relative abundance of fish for overall open water is marked with O.

## Abbreviations

Environmental DNA metabarcoding, eDNA; Traditional survey method, TSM; Double distilled H_2_O, ddH_2_O; Actinopterygii 16s, Ac16s; Polymerase Chain Reaction, PCR; Ribosomal Database Project, RDP; Nucleotide Sequence Database, NT; Operational taxonomic units, OTUs.
