# Supplementary material for: Comparison of Environmental DNA Metabarcoding and a Traditional Survey Method for Assessing Fish Diversity and Distribution Along Salinity Gradient in an Urban Brackish Reservoir, China
Source: Biology (Basel). 2024 Nov 15;13(11):930. doi: 10.3390/biology13110930 (PMC11592076; doi:10.3390/biology13110930)
Supplement: Supplementary file 1 [file biology-13-00930-s001.zip › biology-3251878-Supplementary.pdf]

Comparison of Environmental DNA Metabarcoding and a Traditional Survey Method for Assessing Fish Diversity and Distribution Along Salinity Gradient in an Urban Brackish Reservoir, China

Xu Wang [1], Jiaqiao Wang [1], Lin Lin [1], Liangmin Huang [1], Kai Liu [1], Guangjie Dai [2], Qianwen Cai [2], Jun Li [1], Shilong Feng [1], Guangzhao Wang [1], Yapeng Hui [1], Longhui Qiu [3] and Fenfen Ji [1,*]

[1] Fisheries College, Jimei University, Xiamen 361021, China; 202412951039@jmu.edu.cn (X.W.); skyofstar1@jmu.edu.cn (J.W.); 202411710015@jmu.edu.cn (L.L.); lmhuang@jmu.edu.cn (L.H.); liukai1218@jmu.edu.cn (K.L.); lijun1982@jmu.edu.cn (J.L.); 202312951064@jmu.edu.cn (S.F.); 202221063031@jmu.edu.cn (G.W.); 202412951041@jmu.edu.cn (Y.H.)

[2] Agriculture, Rural and Water Resources Bureau of Jimei District, Xiamen 361022, China; dgjdgj14@sina.com (G.D.); baicai77@sina.com (Q.C.)

[3] Engineering Research Center of Green development for Conventional Aquatic Biological Industry in the Yangtze River Economic Belt, Ministry of Education, College of Fisheries, Huazhong Agricultural University, Wuhan 430070, China; longhui@webmail.hzau.edu.cn

* Correspondence: 202261000140@jmu.edu.cn; Tel.: +86-0592-6181054

Table S1. species detected in the the Xinglinwan Reservoir using eDNA and TSM.

Species	Genus	Family	Order	eDNA meta	Traditional
Oreochromis niloticus	*Oreochromis*	Cichlidae	Cichliformes	+	+
Clupanodon thrissa	*Clupanodon*	Clupeidae	Clupeiformes	+	+
Mugil cephalus	*Mugil*	Mugilidae	Mugiliformes	+	+
Megalops cyprinoides	*Megalops*	Megalopidae	Elopiformes	+	+
Monopterus albus	*Monopterus*	Synbranchidae	Synbranchiformes	+	+
Lateolabrax japonicus	*Lateolabrax*	Lateolabracidae	Perciformes	-	+
Hypophthalmichthys molitrix	*Hypophthalmichthys*	Xenocyprididae	Cypriniformes	+	-
Homatula laxiclathra	*Paracobitis*	Nemacheilidae	Cypriniformes	+	-
Cyprinus carpio	*Cyprinus*	Cyprinidae	Cypriniformes	+	-
Tridentiger bifasciatus	*Tridentiger*	Gobiidae	Gobiiformes	+	-
Hemiculter leucisculus	*Hemiculter*	Xenocyprididae	Cypriniformes	+	-
Hemiculter bleekeri	*Hemiculter*	Xenocyprididae	Cypriniformes	+	-

Note: TSM and eDNA represent the traditional survey method and eDNA metabarcoding, respectively. “-” indicates species absent fromTSM and eDNA; “+” indicates species present from TSM and eDNA

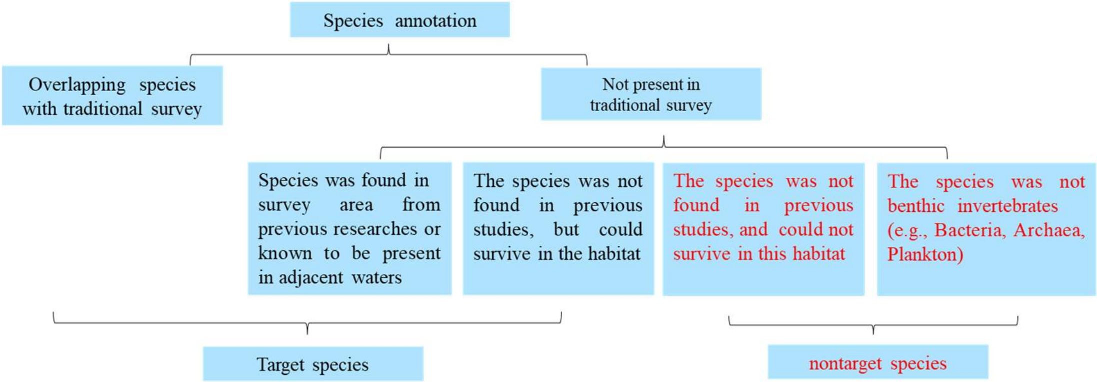

Figure S1. Species-filtering steps identifying target species.

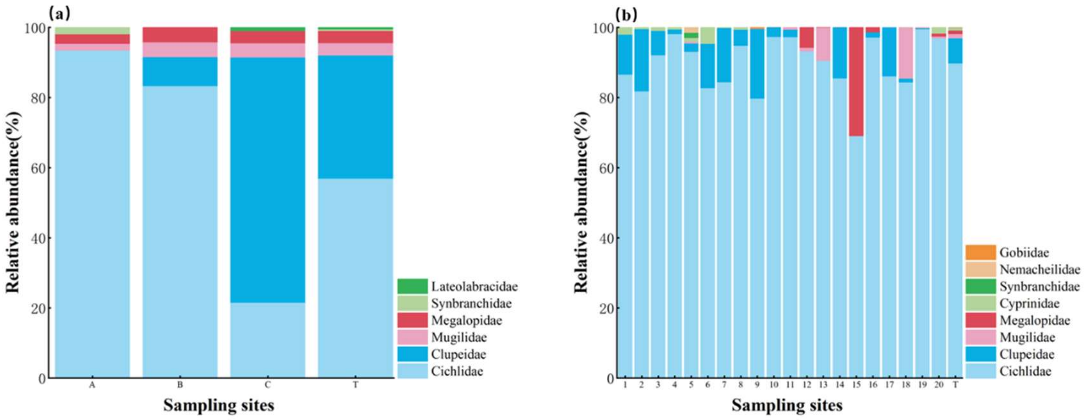

Figure S2. Relative abundance (%) of fish at the family level with TSM (a) or eDNA (b) in per sampling site.

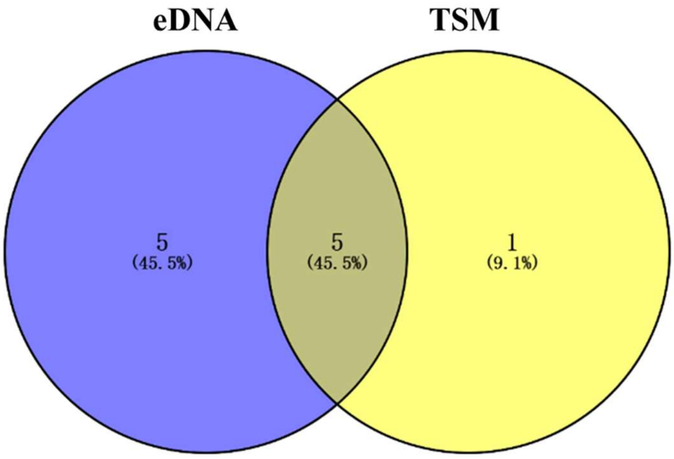

Figure S3. Species richness of fish identified using the TSM and eDNA for the open water sampling sites (sites 5, 7, 11, 13, 14, and 16).

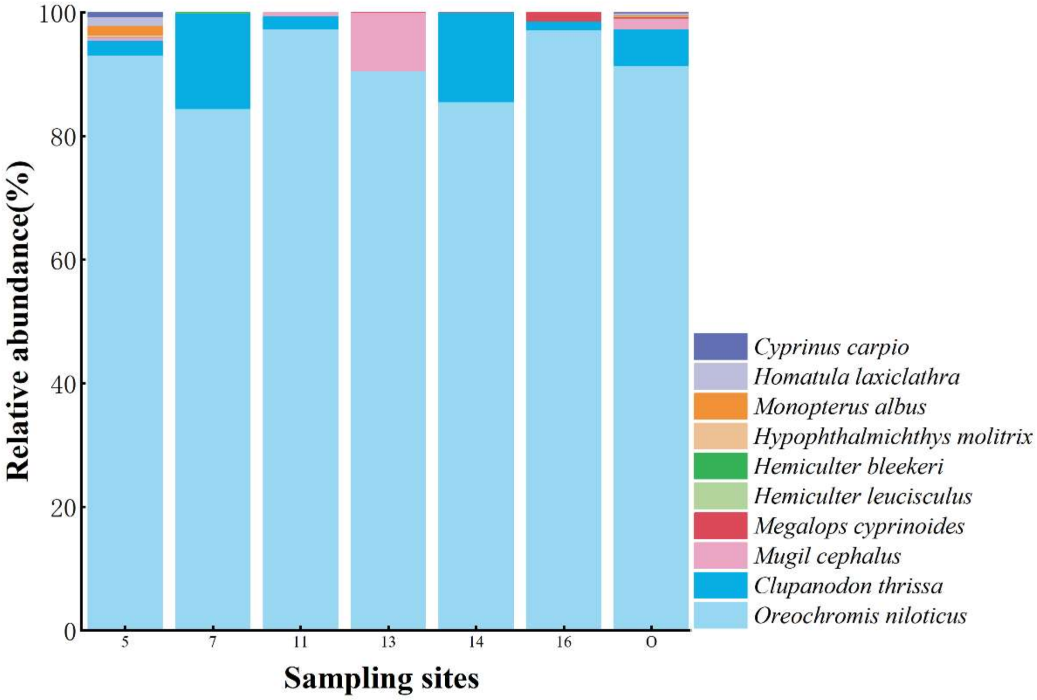

Figure S4. Relative abundance (%) of fish at the species level with eDNA for the open water sampling sites (sites 5, 7, 11, 13, 14, and 16). The relative abundance of fish for overall open water is marked with O.
